# Two remarkable new species of Penicillata (Diplopoda, Polyxenida) from Table Mountain National Park (Cape Town, South Africa)

**DOI:** 10.3897/zookeys.156.2211

**Published:** 2011-12-20

**Authors:** Monique Nguyen Duy–Jacquemin, Charmaine Uys, Jean-Jacques Geoffroy

**Affiliations:** 1 Museum National d’Histoire Naturelle, Département Systématique et Evolution, Section Arthropodes, UMR7205 OSEB, CP 53, 57 rue Cuvier, 75231 PARIS cedex 05, France; 2 Zoology Department, University of Cape Town, Private Bag X3, Rondebosch 7701, South Africa; 3 Muséum National d’Histoire Naturelle, Département Ecologie & Gestion de la Biodiversité, UMR 7204 CERSP, Equipe EvolTrait, 4 avenue du Petit Château, F–91800 Brunoy, France

**Keywords:** Diplopoda, Polyxenidae, Synxenidae, *Propolyxenus*, *Phryssonotus*, new species, scales, barbate trichomes, postembryonic development, South Africa

## Abstract

Two new species of the families Polyxenidae and Synxenidae, are described from Table Mountain National Park, South Africa. *Propolyxenus squamatus* **sp. n.** (Polyxenidae) has tergites I–X mostly covered by scale–shaped trichomes directed caudally, a character previously known only in Synxenidae. The structure of scale–shaped dorsal trichomes is different to that of the scales in *Phryssonotus* and *Condexenus* species. *Phryssonotus brevicapensis* **sp. n.** (Synxenidae) is the only known species of the genus *Phryssonotus* having 11 tergites, (including collum and telson) and 15 pairs of legs, as in *Condexenus biramipalpus* Nguyen Duy–Jacquemin, 2006. These two species therefore appear to occupy an intermediate position between *Phryssonotus* (12 tergites) and Polyxenoidea (maximum of 11 tergites).

## Introduction

Two new species of Penicillata from Table Mountain National Park (near Cape Town), South Africa, belonging to two different families, were collected in the same biotope: leaf litter of felled pine and fynbos.

The first species, represented by five specimens, belongs to the family Polyxenidae and the genus *Propolyxenus* Silvestri, 1948, created for *Propolyxenus aegeus* Silvestri, 1948 from Rhodes (Pelecano) (Silvestri 1948). Silvestri distinguished *Propolyxenus*, with three transversal rows of trichomes per tergite, from the genus *Polyxenus* Latreille, 1803 which has only two rows. Condé and Nguyen Duy–Jacquemin (2008) united *Propolyxenus*, *Polyxenus* and *Typhloxenus* Condé, 1954 in the subfamily Polyxeninae Lucas, 1840, based on the structure of the telson. Silvestri’s brief description was revised after an examination of diagnostic features of two adult syntypes of each sex by Short and Huynh (2010), who also gave a key to the species for the first time.

The second species, represented by 20 specimens, belongs to the family Synxenidae and the genus *Phryssonotus* Scudder, 1885 (a replacement for the preoccupied name *Lophonotus* Menge, 1854), whose type species is *Lophonotus hystrix*, a fossil found in Eocene Baltic amber (Menge in Koch and Berendt 1854, Scudder 1885, Condé 1954). Silvestri (1900) created the genus *Synxenus* Silvestri, 1900 for *Synxenus orientalis* Silvestri, 1900 from Uruguay. Later on, Silvestri (1923) transferred to this genus *Polyxenus platycephalus* Lucas, 1846, from North Africa, Spain and Italy (Rasnitsyn and Golovatch 2004), and described *Synxenus capensis* Silvestri, 1923 from southern Africa (Stellenbosch) and *Synxenus novaehollandiae* Silvestri, 1923 from Australia (Mt Lofty, South Australia). The genus *Synxenus* was synonymized with *Phryssonotus* by Condé (1954). Silvestri’s (1923) identification key included four of the six extant species now known; the main distinguishing characters in the key were the number of ocelli and barbate trichomes (2–6) arranged in a row near the anterior trichobothrium with a short funicule. Short and Huynh (2006) redescribed *Phryssonotus novaehollandiae* and observed 11 ocelli from larva VI to adults. This was an improvement to Silvestri’s key which nevertheless still concerned only four species. The two new species described in the present work show some additional and easily identifiable characters.

Abbreviations used:

**MNHN**	Muséum National d’Histoire Naturelle, Paris, France

**SEM**	Scanning Electron Microscopy

**pl**	pairs of legs

## Material and methods

The material serving as the basis for the present work was obtained by hand collecting, pitfall trapping and litter sampling in pine and fynbos areas in Table Mountain National Park, South Africa (by Charmaine Uys). The material is preserved in 70% ethanol. The bulk of this material, including the holotypes and several paratypes, has been deposited in MNHN.

For light microscopy, the specimens are mounted on slides in “Baume de Marc André”. SEM micrographs were taken using a scanning electron microscope at the Zoology Department, University of Cape Town.

## Systematics

### Class Diplopoda de Blainville in Gervais, 1844

**Subclass Penicillata Latreille, 1831**

**Order Polyxenida Verhoeff, 1934**

**Superfamily Polyxenoidea Lucas, 1840**

**Family Polyxenidae Lucas, 1840**

**Subfamily Polyxeninae Lucas, 1840**

#### Genus Propolyxenus

Silvestri, 1948

Propolyxenus  Silvestri, 1948
Propolyxenus  Silvestri, 1948: Nguyen Duy–Jacquemin and Geoffroy 2003: 100.
Propolyxenus  Silvestri, 1948: Short and Huynh 2010: 13.

##### Remarks.

The genus is typical of the subfamily Polyxeninae, due to the structure of the telson, but shows more than 2 transverse rows of barbate trichomes on each tergite.

##### Propolyxenus squamatus

Nguyen Duy–Jacquemin, Uys & Geoffroy sp. n.

urn:lsid:zoobank.org:act:B4809CFA-3D81-413E-8F07-ADEEE3398883

http://species-id.net/wiki/Propolyxenus_squamatus

**Figure 1.**
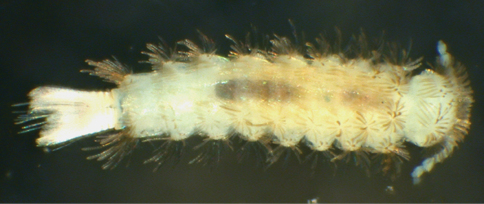
*Propolyxenus squamatus* sp. n. subadult male, habitus, dorsal view, body length: 2.50 mm. (Photograph by M. Judson).

Figs 1 [Fig F2] [Fig F3] [Fig F4] –5 

###### Type material.

South Africa, Cape Town, Table Mountain National Park. Cecilia, Rooikat, site 12, felled pine, altitude 300 m, 33°59'43S, 18°25'22E, 4/X/2008, holotype adult female (no. 4); Cecilia, Rooikat, site 9, Afrotemperate forest, altitude 400 m, 33°59'34S, 18°25'12E, 4/X/2008, paratype adult female (no. 3); other paratypes: Kirstenbosch, Afrotemperate forest, site 5, altitude 400 m, 33°58'55S, 18°25'25E, 12/IX/2008, subadult female (12 pl) (no. 2); Cecilia, Spilhaus, Afrotemperate forest, site 13, altitude 400 m, 33°59'43S, 18°25'05E, 18/X/2008, larva with 8 pl (no. 5), all collected from leaf litter by Charmaine Uys and mounted on slides (MNHN).

###### Other material examined (non–type).

Cecilia, Spilhaus, Fynbos, leaf litter, site 14, 33°59'53"S, 18°24'52"E, altitude 520 m, 18/X/2008, subadult male (12 pl) (no. 6), used for SEM.

###### Etymology.

The specific name refers to the scale–shaped tergal trichomes.

###### Diagnosis.

Differs from all other congeners by the position and structure of the tergal trichomes: these flat scale–shaped trichomes cover the tergites and are different from the barbate trichomes of the lateral tufts, pleurites and head. They are observed for the first time in the family Polyxenidae. As in the family Synxenidae, they lie close to the tergites and are all directed caudally, but differ from those of Synxenidae in their shape and structure.

###### Description of two adult females.

Measurements. Body length (without caudal penicil): 2.50 mm (holotype). Tarsus II length of 13th leg: 100 µm (holotype) and 105 µm (paratype).

**Figure 2. F2:**
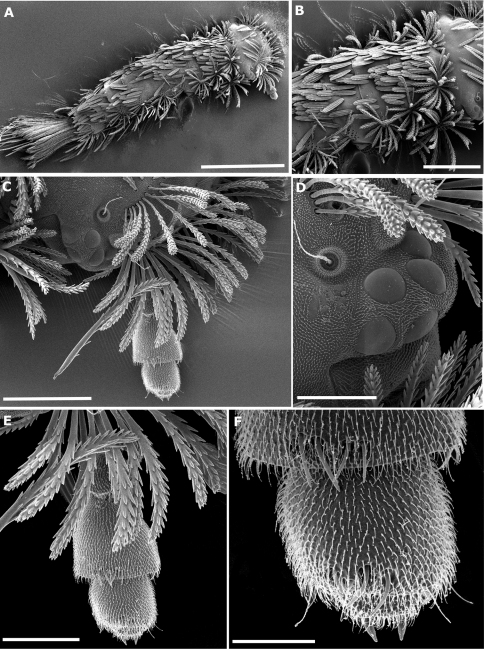
*Propolyxenus squamatus* sp. n. subadult male. **A** habitus, dorsal view **B** collum and tergites II–V, latero–dorsal view **C** right part of head with 2 trichobothria and antenna **D** ocelli and trichobothria **E** 4 last articles of right antenna **F** antennal sensilla on articles VI and VII, apical cones on article VIII. Scale bars: **A** 500 µm **B** 200 µm **C** 100 µm **D** 40 µm **E** 50 µm **F** 20 µm.

**Figure 3. F3:**
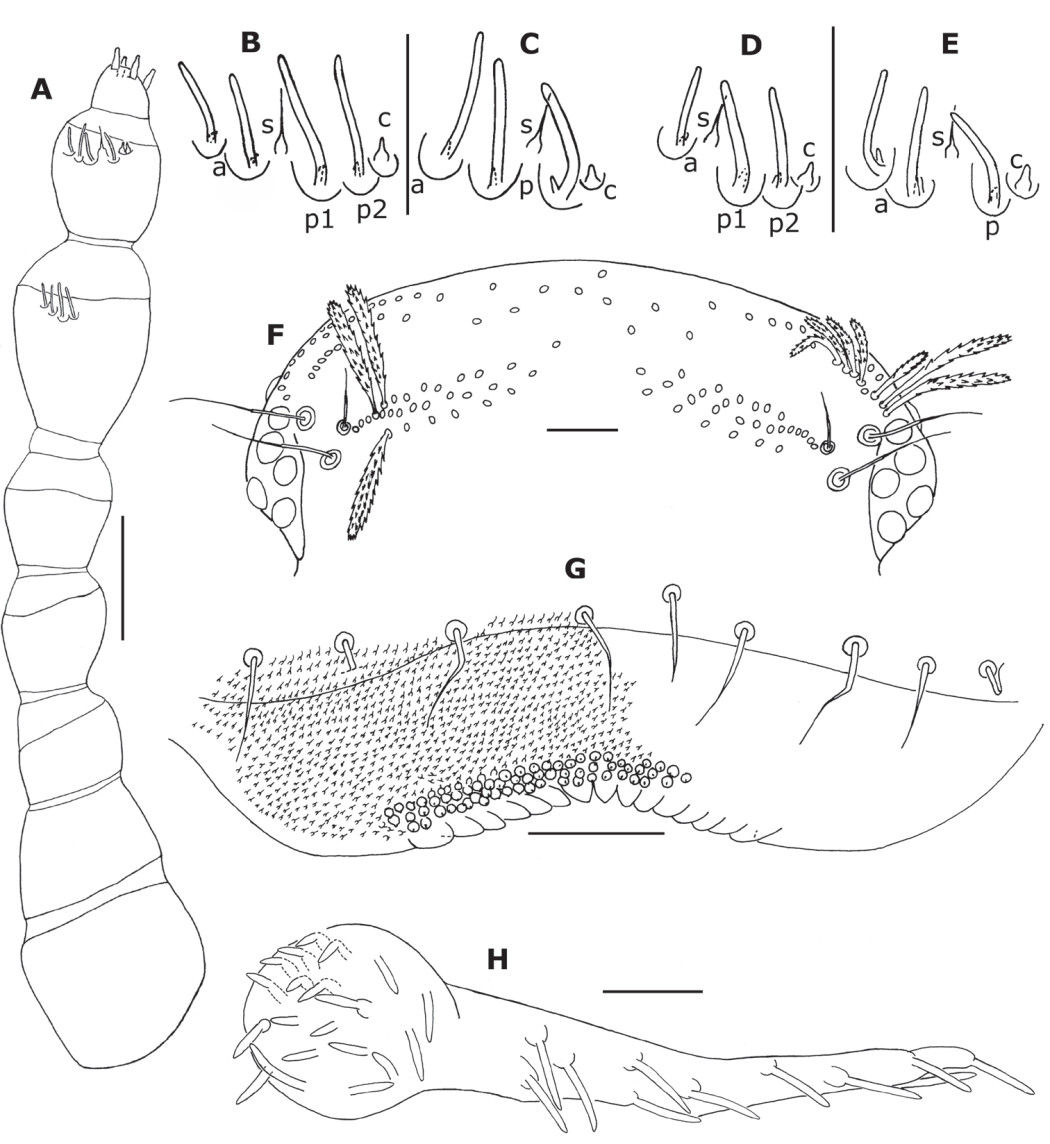
*Propolyxenus squamatus* sp. n. **A** right antenna of holotype female **B, C** sensilla of right antennal articles VI and VII of female paratype (no. 3) **D, E** sensilla of articles VI and VII right antenna of larva with 8 pl (no. 5) **F** vertex of holotype female **G** labrum of holotype female, papillae only represented on right part **H** left palp of gnathochilarium female paratype (no. 3). Abbreviations: **a** anterior basiconic sensillum **c** coeloconic sensillum **p, p1, p2** posterior basiconic sensillum **s** setiform sensillum. Scale bars: **A, F** 50 µm; others, 25 µm.

Head (Fig. 2C). 6 ocelli on each side of which 1 antero–sternal (Figs 2D, 3F). Vertex with 1 pair of posterior tufts of 27+27 (holotype) and 24+25 trichomes (paratype), consisting of 3 rows, middle row with 12–13 trichomes (Fig. 3F); the distance between each tuft is about half their length.

Proportions of antennal articles as in Fig. 3A. Antennal article VI with 4 basiconic sensilla (Figs 2E, F, 3A): 2 anterior (a)shorter and thinner than the 2 posterior ones; the more posterior (p2)slightly thinner than the (p1)(Fig. 3B); 1 setiform sensillum (s) between anterior and posterior ones and 1 posterior coeloconic sensillum (c); antennal article VII with 3 basiconic sensilla, the anterior (a) slightly thinner than the others (Fig. 3C), 1 setifom sensillum (s) between the 2 posterior basiconic sensilla and 1 posterior coeloconic sensillum (c). The right antennal article VII of the holotype has 4 basiconic sensilla and 2 coeloconic sensilla, but this is recognizable as a regenerated antenna (as shown by Nguyen Duy–Jacquemin 1972) with 2 coeloconic sensilla on article VII and none on article VI (Fig. 3A).

3 trichobothria, arranged in a triangle, with the most internal (near posterior tufts of vertex) smaller than the 2 others (Figs 2C, D, 3F). Surface of labrum (Fig. 3G) with numerous small short cuspidate papillae; papillae of anterior 2 to 3 rows larger; 7+8 lamellate teeth on anterior margin (holotype: Fig. 3G), 8+8 (paratype); clypeo–labrum with 9 setae along posterior margin (Fig. 3G). Outer palp of gnathochilarium with 11 or 12 sensilla; middle palp with 19 or 20 sensilla (Fig. 3H).

**Figure 4. F4:**
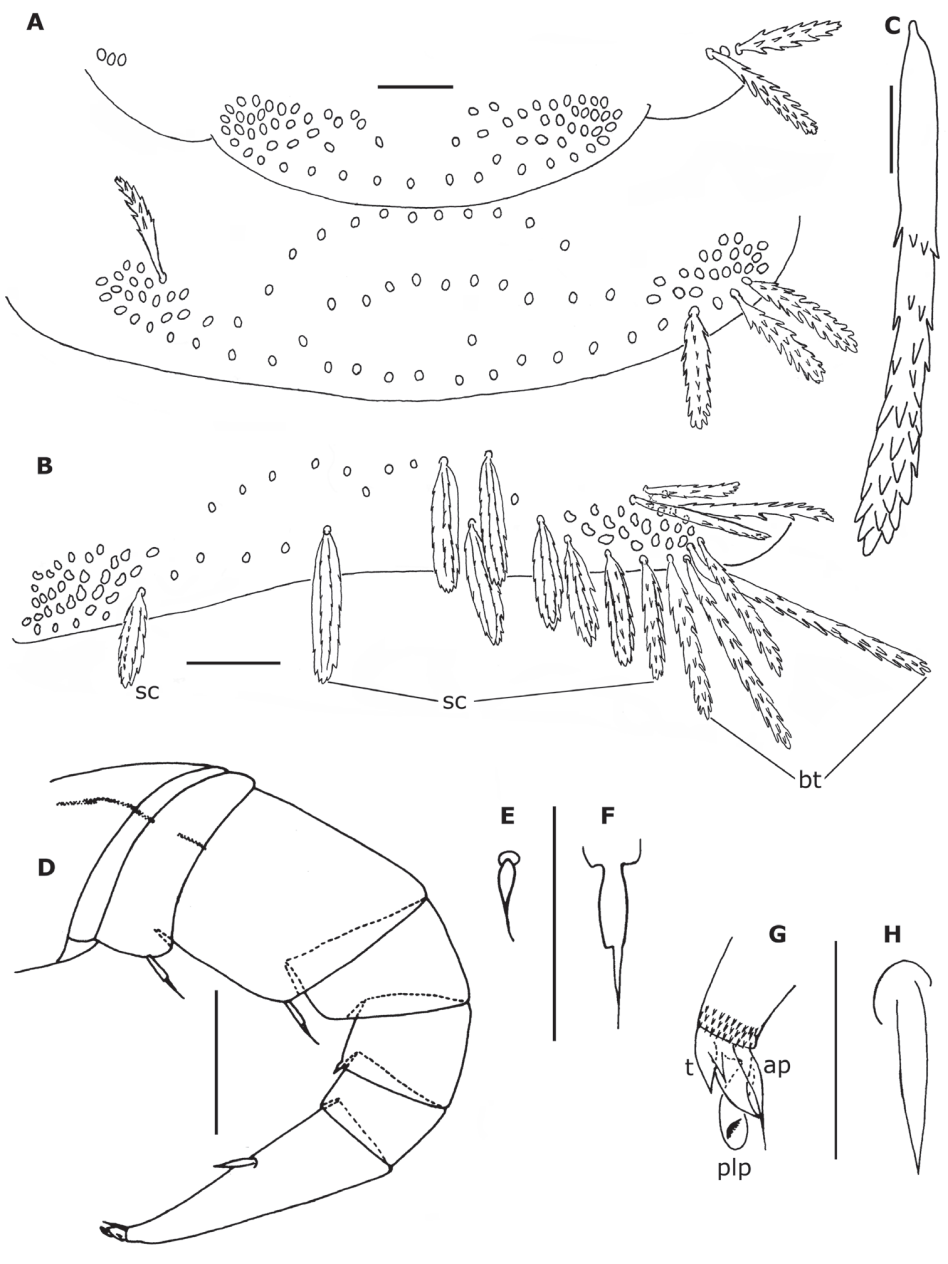
*Propolyxenus squamatus* sp. n. **A** Collum and tergite II of holotype female **B** tergite X of holotype female **C** barbate trichome of the right lateral tuft of tergite VII of holotype female **D** left leg 12 of holotype female **E, F** details of tibial and prefemora setae of the left leg 12 **G, H** telotarsus and tarsal II spine of right leg 13 of female paratype (no. 3). Abbreviations: **ap** anterior process **bt** barbate trichomes **plp** posterior lamellate process **sc** scale **t** latero–anterior and posterior teeth. Scale bars: **C, E–H** 25 µm; others, 50 µm.

**Figure 5.**
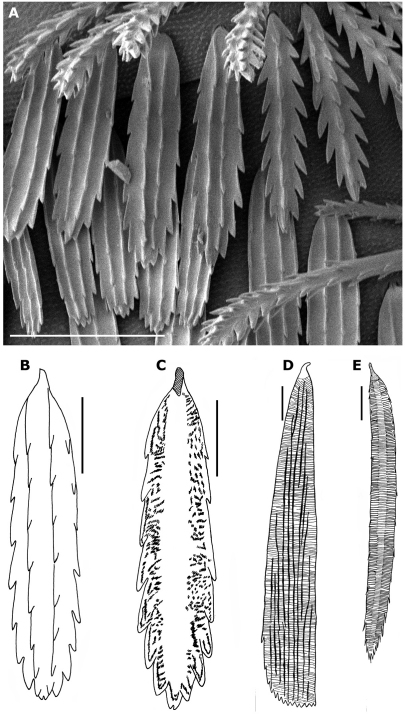
*Propolyxenus squamatus* sp. n. **A** tergal scales **B, C** scale of tergite VII of paratype female. external and internal views respectively **D** scale of tergite VIII of *Phryssonotus capensis* male with 12 pl from Mtuzini, Natal **E** scale of posterior row of tergite VIII of the holotype *Condexenus biramipalpus*. **D** and **E** modified after Nguyen Duy–Jacquemin 2006. Scales bars: **A** 50 µm **B–E** 25 µm.

Trunk (Fig. 2A): On each tergite (except collum, tergite X and telson) the trichomes are arranged in 3 rows and 2 lateral tufts (Figs 2B, 4A, B); each paired tuft connected by posterior and anterior rows of trichomes; middle row with more spaced trichomes (Fig. 4A). There are 2 types of trichomes. The flat trichomes, referred to as scales (sc) of the 3 rows are wider than barbate trichomes of lateral tufts (bt) and their shape and structure are different (Figs 2B, 4B, 5A–C); they look like the scales of Synxenidae by their position: all are directed caudally and cover the tergites I (with only a posterior row) to X (Figs 4A, B). Their structure is different from scales of Synxenidae (Figs 5A–E). The trichomes of the lateral tufts are longer and arranged in a bunch (Figs 2B, 4A, B (bt), C). Lateral protuberance of tergite I with 3 barbate trichomes (Fig. 4A).

Legs (Figs 4D–H): Naming of leg segments is after Brolemann (1935). Coxae I with 1 seta and coxae II with 2 setae; all other coxae without seta. All trochanters and prefemora with 1 seta; these setae having an oval base furnished with acute process at apex (Fig. 4F). All tibias (except 13) have 1 small seta tapered apically as shown in Figs 4D, E; other articles without seta. Tarsus II spine (Fig. 4H) longer than telotarsus (Fig. 4G): length of spine to claw ratio about 1.80. Telotarsus bearing an anterior process (ap) with a spinous projection longer than claw, 2 latero–anterior and posterior spiniform processes (t), posterior larger than anterior; posterior lamellar process (plp) thickened and basally pleated (Fig. 4G).

Telson (Figs 1, 2A): typical of genera *Propolyxenus*, *Polyxenus*, *Typhloxenus* (subfamily Polyxeninae). 21 (holotype) and 25 (paratype) dorsomedian barbate trichomes on caudal penicil. Hooked trichomes with 3 or 4 hooks.

###### Description of subadult female, 12 pl (no. 2).

Measurements. Body length (without caudal penicil): 2.40 mm. Caudal penicil length: 0.60 mm. Tarsus II length of 12th leg: 112 µm.

Head: 6 ocelli on each side. Antennal article VI with 3 basiconic sensilla (Fig. 3D); antennal article VII with 3 basiconic sensilla (Fig. 3E). Surface of labrum with numerous small short cuspidate papillae; papillae of anterior 2 rows larger; 9+9 lamellate teeth at anterior margin. Outer palp of gnathochilarium with 11 sensilla; middle palp with 19 sensilla.

Trunk: Scales on tergites I-IX. Lateral protuberances of tergite I with 3 barbate trichomes.

Legs: Coxae I with 1 seta and coxae II with 3 setae; all other coxae without setae. All trochanters and prefemora with 1 seta. All tibias (except 11 and 12) have 1 small seta tapered apically; other articles without seta. Telotarsus bearing an anterior process with a spinous projection longer than claw, 2 latero–anterior and posterior spiniform processes; posterior lamellar process thickened and basally pleated.

Telson: 22 dorsomedian barbate trichomes of caudal penicil. Hooked trichomes with 3 or 4 hooks.

###### Description of a larva 8 pl (no. 5).

Measurements. Body length (without caudal penicil): 1.80 mm. Tarsus II length of 8th leg: 110 µm.

Head: 6 ocelli on each side. Vertex with 1 pair of posterior tufts of 20+19 trichomes consisting of 3 rows, the middle row with 10 trichomes. Antennal article VI with 3 basiconic sensilla: 1 anterior shorter and thinner than the 2 posterior ones; the more posterior slightly thinner than the other; 1 setiform sensillum between anterior and posterior basiconic sensilla and 1 posterior coeloconic sensillum; antennal article VII with 3 basiconic sensilla, the anterior slightly thinner than the others, 1 setiform sensillum between the 2 posterior basiconic sensilla and 1 posterior coeloconic sensillum. 3 trichobothria, arranged in a triangle, with the most internal smaller than the 2 others. Surface of labrum as in adult females; clypeo–labrum with 10 setae along posterior margin. Outer palp of gnathochilarium with 12 sensilla.

Trunk: Trichomes arranged in 2 lateral tufts with 19 to 25 barbate trichomes connected by 3 rows of scales on tergites III–V and only 2 on other tergites. The tergites II–V have 22 to 31 scales, the collum 12 and the tergite VII 18. Lateral protuberance of tergite I with 3 (left) and 2 (right) barbate trichomes.

Telson: 18 dorsomedian barbate trichomes of caudal penicil. Hooked trichomes with 3 hooks (rarely 2 and 4).

###### Discussion.

*Propolyxenus squamatus* sp. n. is strongly distinguished from other species of the genus by the shape of the trichomes covering the tergites. Compared with the most closely related species *Propolyxenus lawrencei* Condé, 1949, from Natal (Champagne Castle, Drakensberg Mountains, alt. 6000 ft.), *Propolyxenus squamatus* sp. n. shares the following characters: 6 ocelli; internal trichobothrium shorter than the other 2; number and shape of sensilla on antennal articles VI and VII (Condé 1949, p. 125–126, 1959); surface of the labrum with numerous papillae, the 2 or 3 anterior rows larger.

The new species shows the following important differences from *Propolyxenus lawrencei*:

Position and structure of trichomes on tergites: on each tergite (except collum and telson) the trichomes are arranged in 3 rows and 2 lateral tufts; each paired tuft being connected by posterior and anterior rows of trichomes; the middle row has more spaced trichomes. In *Propolyxenus lawrencei*, the trichomes are arranged in 3 or 4 irregular rows, forming 2 elongated lateral areas, slightly separated by a narrow medial space.

There are two types of trichomes: the trichomes of the three rows are wider and flatter than the trichomes of the lateral tufts, pleurites and head, and their shape and structure are different, being observed in the family Polyxenidae for the first time. They can be compared to the scale–shaped trichomes of Synxenidae: the trichomes of the rows are all directed caudally and cover the posterior half of tergites II–X and their internal structure is reinforced by differently distributed chitinous elements (Figs 5C–E). The lateral trichomes are longer and arranged in lateral tufts. It is remarkable that the barbate trichomes of *Propolyxenus squamatus* sp. n. show a progressive transformation into scale–shaped trichomes in the posterior row of the tergite, representing a transition between the two types of trichomes as if, during the course of evolution, the former trichomes had changed into scale–shaped trichomes. These scale–shaped trichomes are thought to protect the animals from desiccation, abundant rain or other environmental disturbances.

In a key of the genus *Propolyxenus*, *Propolyxenus squamatus* sp. n. would be easily distinguished from all other congeners as is only species with scale-like trichomes. The other species of *Propolyxenus* are more difficult to identify using morphological characters such as the number of ornamental trichomes or coxal glands of the males. For instance, both *Propolyxenus patagonicus* (Silvestri, 1903) and *Propolyxenus australis* Short and Huynh, 2010, bear four pairs of coxal glands (cf. Condé and Massoud 1974, p. 227 for *Propolyxenus patagonicus*) contrary to the first tentative key proposed by Short and Huynh (2010 p. 15). There is a difficulty in the limited nature of keys based (even partly) on characters such as coxal glands, requiring collection of adult males. More appropriate characters need to be determined for a more robust key for the genus.

### Superfamily Synxenoidea Silvestri, 1923

**Family Synxenidae Silvestri, 1923**

#### Genus Phryssonotus

Scudder, 1885

Synxenus  Silvestri, 1900
Kubanus  Attems, 1926
Koubanus  Attems, 1928
Schindalmonotus  Attems, 1926
Lophonotus  Menge, 1854, preoccupied, non Stephens, 1829
Kaubanus  (sic) Attems, 1929, misprint by Jones 1937
Schindelmonatus  (sic) Attems, 1929, misprint by Jones 1937

##### Remarks.

The genus *Phryssonotus* is characterised by the tergites having dark striated, scale-shaped trichomes directed caudally, all the others being long, dark barbate trichomes; trichomes A and B on head close to trichobothria, one of them being shorter and different from the two others.

##### Phryssonotus brevicapensis

Nguyen Duy–Jacquemin, Uys & Geoffroy sp. n.

urn:lsid:zoobank.org:act:DADD9CDA-BB36-491F-85BB-FE609759F2E9

http://species-id.net/wiki/Phryssonotus_brevicapensis

**Figure 6.**
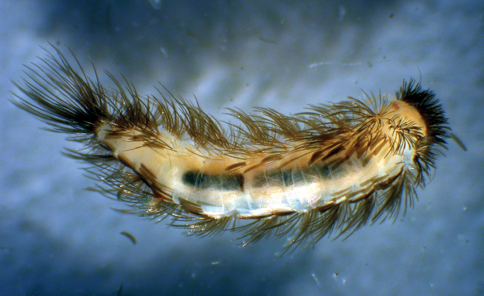
*Phryssonotus brevicapensis* sp. n, habitus, dorsal view, body length: 5 mm. (Photograph by M. Judson).

Figs 6 [Fig F7] [Fig F8] –9 

###### Type material.

South Africa, Cape Town,Table Mountain National Park. Tokai S, site 30, Fynbos, altitude 310 m, 34°04'01S, 18°24'03E, leaf litter, 24/XI/2008, holotype adult male (no. 16a) and 1 paratype adult male (no. 16b) (MNHN). Other paratypes: adult male (no. 1) (MNHN), Newlands, site 4, pine plantation, altitude 260 m, 33°58'24S, 18°26'27E, sugar–baited ant trap, 15/I/2009; adult female (no. 20) (MNHN), Tokai S, site 31, pine plantation, altitude 300 m, 34°03'54S, 18°24'10E, decaying log, 19/I/2009; adult female (no. 11) (MNHN), Constantia Nek, site 19, felled pine, altitude 330 m, 34°00'20S, 18°24'45E, pitfall trap, 02/II/2009; male with 14 pl (subadult) (no. 8) (MNHN), Cecilia, Spilhaus, site 16, felled pine, altitude 470 m, 34°00'04S, 18°24'46E, pitfall trap, 23/0I/2009; female with 14 pl (subadult) (no. 13) (MNHN) and female with 12 pl (no. 15) (MNHN), Tokai N, site 27, pine plantation, altitude 330 m, 34°02'23S, 18°23'53E, leaf litter, 21/XI/2008; 2 larvae with 10 pl and 8 pl (no. 12) (MNHN), Orange Kloof, site 22, pine plantation, altitude 240 m, 34°00'23S, 18°24'02E, leaf litter, 18/XI/2008. All specimens collected by Charmaine Uys.

1 male with 14 pl (no. 6b) was collected at the same site as a male with 12 pl of *Propolyxenus squamatus* sp. n. (no. 6, used for SEM).

###### Etymology.

Refers to the shorter body length and development compared to the most closely related species, *Phryssonotus capensis*.

###### Diagnosis.

10 ocelli; 5 trichomes B close to the smallest trichobothrium (tc) as in *Phryssonotus capensis*. Differing from *Phryssonotus capensis* with 10 rings in adults instead of 11 (without telson), 15 leg pairs instead of 17, and the attendant shorter post–embryonic development. Males with 3 pairs of coxal glands on legs 7–9.

###### Description of adults, males and females.

**Figure 7. F7:**
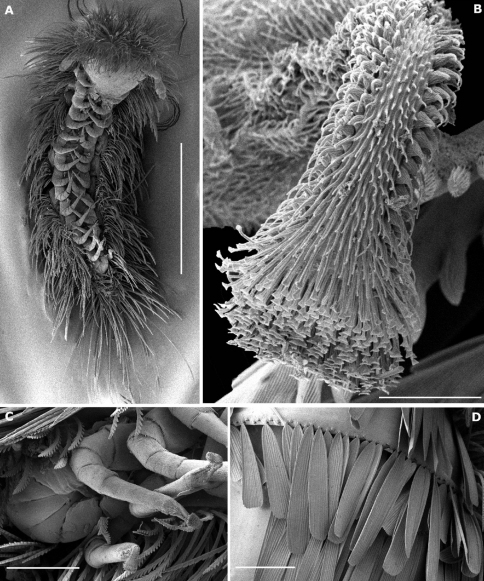
*Phryssonotus brevicapensis* sp. n. **A** habitus, adult male, ventral view showing 15 pl **B** detail of a palette of left leg 15 **C** postero–ventral view showing leg pairs 14 and 15 terminating in palettes **D** detail of scales arranged along posterior margin of tergite. Scale bars: **A** 1 mm **B** 10 µm **C, D** 100 µm.

Measurements. Body length (without caudal penicil): 4.00–4.50 mm; caudal penicil length: 0.90–1.00 mm (Figs 6, 7A).

**Figure 8. F8:**
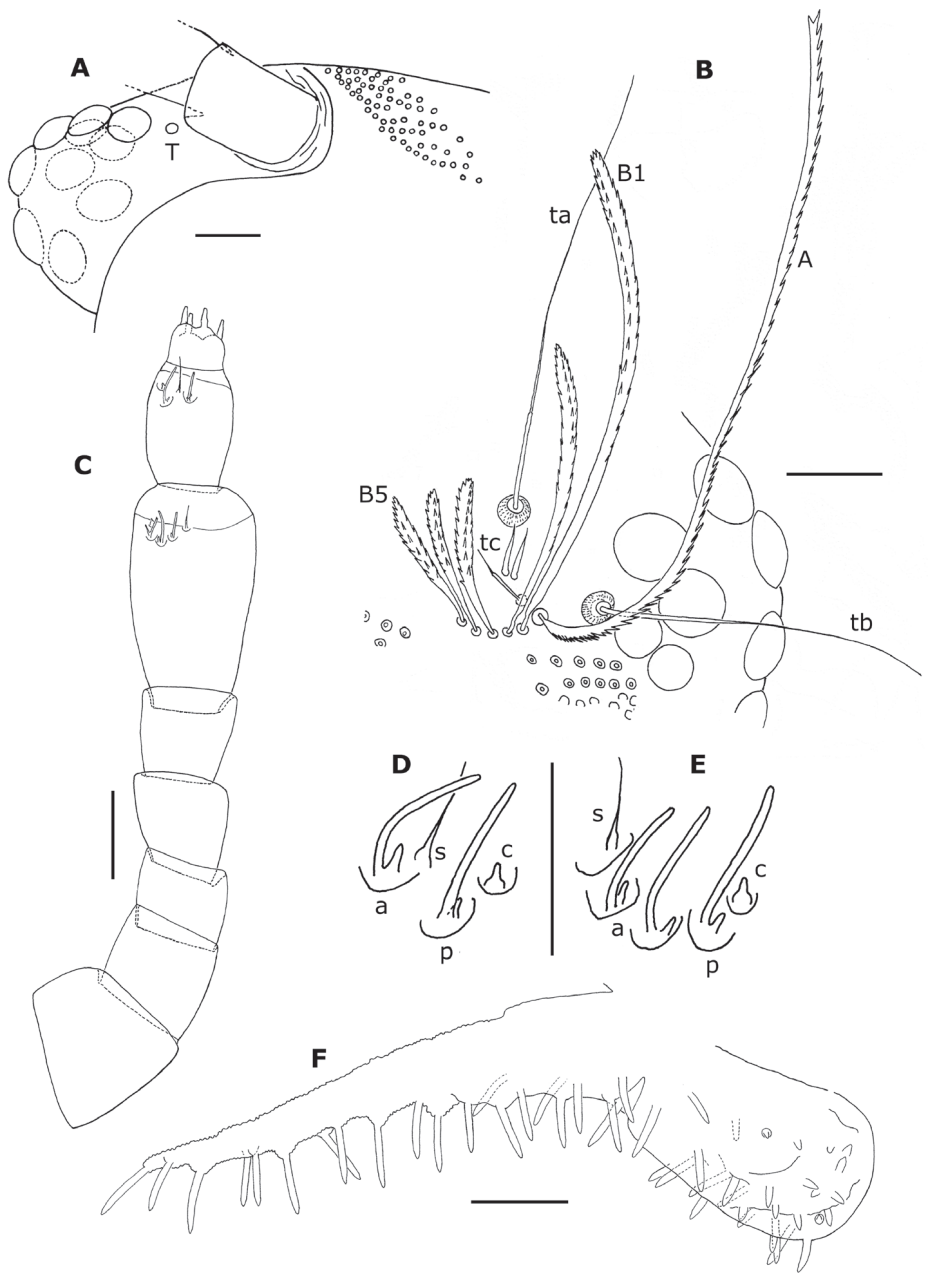
*Phryssonotus brevicapensis* sp. n. **A** right ventral part of head of female adult (no. 20) showing the 10 ocelli, dorsal ones shown with dotted lines **B** right part of the head of holotype showing position of trichobothria ta, tb and tc, long frontal trichome A and short trichomes B1–B5 (only some ocelli drawn) **C** left antenna of holotype; the posterior sensillum is abnormally bifurcated on article VI **D, E** antennal sensilla on articles VII and VI of right antenna of holotype **F** right palp of gnathochilarium of holotype. Abbreviations: **a** anterior basiconic sensillum **c** coeloconic sensillum **p** posterior basiconic sensillum **s** setiform sensillum; T, Tömösvary’s organ. Scale bars: **A, B, C** 50 µm; others, 25 µm.

Head with 10 ocelli on each side (Fig. 8A); 3 trichobothria with the anterior 1 (tc) possessing a much shorter sensory hair than the other 2 (ta and tb). 5 short frontal trichomes B1–B5 and 1 long, curving trichome A (Fig. 8B).

Proportions of antennal articles as in Fig. 8C. Antennal article VI with 3 basiconic sensilla (Fig. 8C, E): 2 anterior (a), which are shorter and slightly thinner than the posterior one (p); 1 anterior setiform sensillum (s) and 1 posterior coeloconic sensillum (c); antennal article VII (Fig. 8D) with 2 basiconic sensilla, the anterior (a) slightly shorter than the posterior one (p), 1 setifom sensillum (s) between the 2 basiconic sensilla and 1 posterior coeloconic sensillum (c).

Surface of labrum with numerous, small, short cuspidate papillae; papillae of anterior 3 or 4 rows larger, the size of the following papillae decreasing progressively, the smaller ones in the posterior third; about 30 lamellate teeth at anterior margin. Clypeo–labrum with ca. 10 setae, about 3/4 maximum width of labrum. Lateral expansions of gnathochilarium about twice as long as diameter of middle palp, with 21–25 sensilla, middle palp with 26–29 sensilla, of which antero–medial sensilla shorter than the others (Fig. 8F).

Trunk of adults with 11 tergites (including collum and telson) and 15 pairs of legs (Fig. 7A). Collum with 2 medial, separate oval clusters comprising 80–90 barbate trichomes and a lateral group of 8–14 barbate trichomes. Tergites II–X with submedial and posterior rows of scale–shaped trichomes directed caudally (Fig. 7D), the posterior row arranged along the posterior margin of the tergite; 1 area of aligned barbate trichomes at end of each row, except on tergite II where are 35–40 barbate trichomes arranged, on each side, in 2 diagonal lines above the first scale–row; 2 short rows of barbate trichomes at end of submedial scale–row and 4 (sometimes 5 on tergite II and X) short rows of barbate trichomes at end of posterior scale–row. The number of scales by row ranges from 29–62 on tergites II–X.

**Figure 9.**
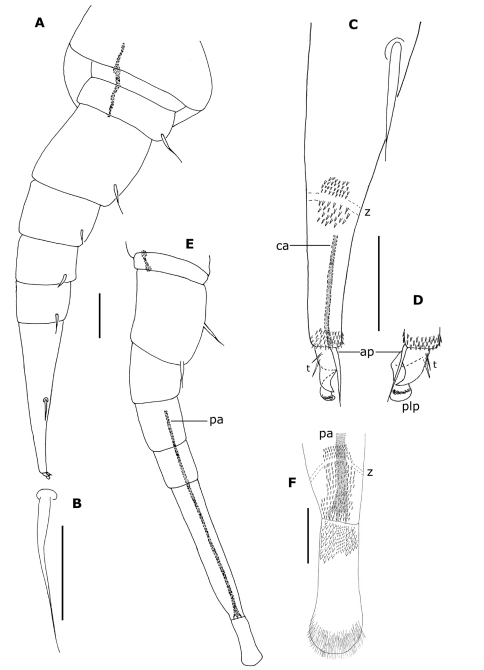
*Phryssonotus brevicapensis* sp. n., adult male (no. 1). **A** right leg 12 **B** seta of prefemur of right leg 12 **C** distal part of tarsus II of the right leg 12 **D** telotarsus of left leg 12 **E** right leg 15 **F** distal part of tarsus II and palette of right leg 15. Abbreviations: **ap** anterior process **ca** apodeme of claw **pa** apodeme of palette **plp** posterior lamellate process **t** latero–anterior and posterior teeth **z** smooth area. Scale bars: **A, E** 50 µm; others, 25 µm.

Legs short (Fig. 9A), with 8 articles except on legs 1, 14 and 15; last 2 pairs (14–15) without telotarsus, tarsus II terminated in palettes (Figs 7B, C; 9E, F); palettes covered by numerous cuticular setae of different types (Figs 7B; 9F); apodeme of palettes (pa) and claws (ca) extending into distal part of trochanter and linked to the flexor unguiculi muscle, which allows a great flexibility of the palette of the leg pairs 14 and 15, as well as to the claws of legs 1–13, in accordance with the description given by Manton (1956) for *Polyxenus lagurus*. Legs 1–13 with each trochanter, prefemur (Fig. 9B), femur, tibia and tarsus I bearing a single long and very fine seta; seta of second tarsus longer than claw (Fig. 9C). Legs 14 and 15 with only 2 setae on prefemur (Fig. 9E). Telotarsus bearing an anterior process (ap) with spinous projection longer than claw; lamellate process (plp) thickened and basally pleated; claw with 2 subequal, strongly pointed latero–anterior and posterior teeth (Fig. 9C, D).

Female: large vulval sacs elongated, reaching as far as fourth pair of legs and bearing numerous small setae inserted in parallel circles and sparse longer setae.

Male: all areas of penis with usual thin cuticular setae and about 15 longer setae (holotype). Coxal glands on legs 7–9.

Conical telson with a transverse row of 15 (male no. 1), 10 (male no. 16b) or 14 (female no. 20) scale–shaped trichomes with each lateral end prolonged by barbate trichomes; long barbate trichomes on distal part.

###### Subadults.

1 male, 1 female: Measurements: Body length (without caudal penicil): 3.20 mm (male no. 8) and 3.90 mm (female no. 13); caudal penicil length: 0.90 mm. 14 pairs of legs, the 14th terminating in a palette; 1 pair of appendage–buds on lateral side of anal valves, from which 15th pair of legs will develop, the future adult stadia having the leg pair 15 terminating in palettes. Other characters as in adults, except no scale–row on telson.

Male: Coxal glands on legs 7–9.

Female: large vulval sacs elongated, reaching as far as fourth pair of legs.

###### Stadium VII.

1 female (no. 15) with 12 pl; body length (without caudal penicil): 3.80 mm; caudal penicil length: 0.80 mm; 10 ocelli, vulval sacs elongated, reaching as far as third pair of legs. 2 pairs of external buds.

###### Stadium VI.

1 juvenile male with 10 pl; body length (without caudal penicil): 3.30 mm; 10 ocelli; rudimentary coxal glands on legs 7 and 8. 2 pairs of external buds.

###### Stadium V.

1 larva with 8 pl; body length (without caudal penicil): 2.70 mm; 9 ocelli. 2 pairs of external buds.

###### Discussion.

*Phryssonotus brevicapensis* sp. n. exhibits all the general characters usually present in the family Synxenidae: long and thin dark barbate trichomes all along the body, tergites covered by tergal scale–shaped trichomes that are striated and arranged in 2 transverse rows along all the tergites except the collum; telson subconical; elongated vulvae; and last 2 leg pairs terminating in palettes instead of claws. It also shows the typical structure of the scale–shaped trichomes found in the genus *Phryssonotus*. *Phryssonotus brevicapensis* sp. n. differs from other members of the genus in having 11 tergites and 15 pl; the last 2 pairs (14th and 15th) terminating in palettes; and males with 3 pairs of coxal glands on legs 7–9. These differences are strongly related to biology and development, and justify the creation of a new species. All other species of *Phryssonotus* have 12 tergites and 17 pairs of legs with the last 2 pairs (16th and 17th) terminating in palettes, and males with 3 pairs of coxal glands on legs 9–11. Due to its shorter length and the position of the coxal glands on legs 7–9, it is similar to *Condexenus biramipalpus*. The elongated vulvae (ovipositors) of the females also resemble those of *Condexenus* species in reaching as far as the fourth pair of legs, as opposed to sixth at most in other *Phryssonotus* species.

*Phryssonotus brevicapensis* sp. n. is most closely related to *Phryssonotus capensis*, in having 10 ocelli and 5 short frontal trichomes B1 to B5, *Phryssonotus capensis* has 5–6 trichomes B (Silvestri 1923).

**Figure 10.**
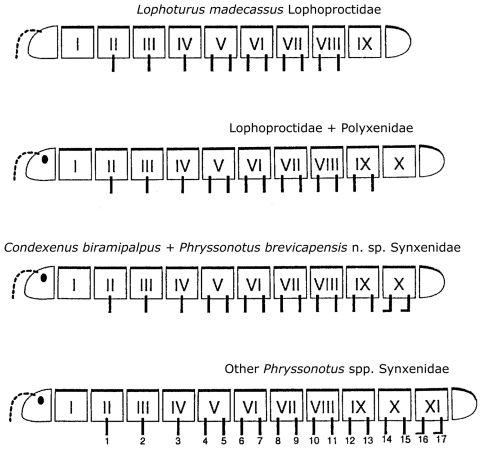
Comparison of segmentation in Polyxenida, corrected and improved after  Nguyen Duy–Jacquemin (2006).

Following the discovery of *Condexenus biramipalpus* from Namibia, it is of great interest to add *Phryssonotus brevicapensis* sp. n. as the second example of reduction of ring and leg number in the family Synxenidae, whose representatives bear the largest number of segments among the Penicillata. This supports the hypothesis of a trend towards a shortened postembryonic development during the course of evolution of Polyxenida (Condé 1969 and Nguyen Duy–Jacquemin 2006). The comparison of the pattern of development is emphasised in the improved scheme (Fig. 10), in which the shortest development pattern is seen in *Lophoturus madecassus* (Marquet et Condé, 1950). The two new species described in the present work strongly support this evolutionary trend among penicillate families.

## Supplementary Material

XML Treatment for Propolyxenus

XML Treatment for Phryssonotus
